# Evaluation of Internet-based pharmaceutical care effect on young and middle-aged patients with hypertension by the principal component analysis and the Markov cohort during COVID-19 pandemic

**DOI:** 10.1186/s12955-023-02168-0

**Published:** 2023-08-18

**Authors:** Xinmei Li, Wenxiu Xu, Xiaofeng Mo, Fan Wu, Minghong Qu, Junxian Ye, Wenxing Wu, Haizhi Li, Te Li

**Affiliations:** 1 Department of Pharmacy, Fuwai Yunnan Cardiovascular Hospital, Shahe North Road, Yunnan Province 650000 Kunming, China; 2 Yunnan Provincial Key Laboratory of Pharmacology, Kunming Medical University, Kunming, China

**Keywords:** Hypertension, Pharmaceutical care, Principal component analysis, Orthogonal partial least-discriminant analysis, The Markov cohort

## Abstract

**Background:**

Pharmaceutical care has the potential to improve hypertension control rates in young and middle-aged patients. Due the COVID-19 epidemic, standard intervention methods may not be applicable. We propose establishing an internet-based pharmaceutical care (IPC) route to improve blood pressure control in young and middle-aged patients with hypertension. An evaluation method based on Principal Component Analysis (PCA) and Orthogonal Partial Least-Discriminant Analysis (OPLS-DA) was established to evaluate the effect of the IPC method.

**Methods:**

1) Internet-based Pharmaceutical care (IPC) was provided by pharmacists mainly using Wechat software for one year after enrollment; 2) PCA and OPLS-DA were applied to analyze questionnaire reliability and data variability; 3) Markov cohort was used to evaluate the IPC effect.

**Results:**

Ninety-seven young and middle-aged patients were enrolled. 96 patients received the IPC. 1) The blood pressure control rate increased to 71.88% after IPC in 96 patients. 2) After conducting PCA and OPLS-DA analysis, 10 questions in the questionnaire were significantly improved after the IPC. 3) Markov cohort results showed that patient survival after 28 cycles was 18.62 years and the quality-adjusted life year (QALY) was extended by 5.40 years. The cumulative cost-effectiveness ratio was ¥87.10 per QALY.

**Conclusions:**

The IPC method could significantly improve the blood pressure control rate of patients. The questionnaire analysis method based on PCA and OPLS-DA is an effective method to evaluate the effect of the IPC method. The Markov cohort showed that the IPC had an effect on blood pressure control rate changes. Patients had a strong willingness to pay for IPC.

**Supplementary Information:**

The online version contains supplementary material available at 10.1186/s12955-023-02168-0.

## Introduction

Long-term treatment of hypertension is widely recognized as a significant public health challenge worldwide due to its high incidence of associated complications [1, 2]. In China, the control rate of drug treatment is 37.6%, with only about 10% for young and middle-aged people [3, 4]. The main cause of poor blood pressure control in this age group might be attributed to poor compliance with medication and fast-paced lifestyles [5–7]. With the rising occurrence of severe hypertension complications (e.g. ischemic stroke, cerebral microbleeds, chronic kidney disease, ischemic colitis) in young and middle-aged patients, prevention of complications and stable blood pressure control (through medication and lifestyle interventions) is important [8–12].

Effective pharmaceutical care, including medication and lifestyle, might be the main measure for controlling blood pressure. A study showed that the control rate was effectively improved (46.9%) after 3 months of pharmacist intervention [12]. A meta-analysis of 69 studies with a total of 11,644 patients showed that pharmaceutical care reduced systolic blood pressure by 8.5 mmHg and diastolic blood pressure by 3.68 mmHg [13]. A study conducted at an emergency hospital on 2,049 patients who were examined pre- and post-intervention using direct observation for the detection of errors found that pharmacist interventions resulted in a significant reduction in the medication error rate from 351 (34.2%) in the pre-intervention phase to 157 (15.3%) in the post-intervention phase (*P* < 0.001) [14]. However, in the COVID-19 pandemic, standard intervention methods such as Smith's patient-centered interviewing of pharmacists are limited [15]. The pandemic may make blood pressure control difficult, leading to a high incidence of cardiovascular events [16]. Therefore, pharmacists urgently need a feasible and cost-effective method to address this issue.

The objective of this study was to achieve sustained and stable control of blood pressure below 120 mmHg through the implementation of IPC intervention. The evaluation of the effect of pharmaceutical care mainly relies on patients' subjective scores of pharmacists, and the verification of objective indicators is lacking. In this study, patients' blood pressure and compliance questionnaires were used to evaluate patients' blood pressure control. The Markov cohort was used to objectively evaluate pharmaceutical care. Our research group has examined the principal component analysis (PCA) and orthogonal partial least-discriminant analysis (OPLS-DA) for the prognosis analysis of inpatients [17, 18]. In this study, PCA and OPLS-DA were used for the first time to analyze an online outpatient follow-up questionnaire, with questionnaire scores serving as indicators of the effectiveness of Internet-based pharmaceutical care (IPC). As hypertension is a chronic disease, short-term results and treatment costs are often closely related to long-term outcomes, quality of life, and treatment costs. By dynamically simulating the progression of chronic disease using Markov models, it is possible to reconcile morbidity, cost, and quality of life for patients [19, 20]. Markov cohort is now widely used in pharmacoeconomics to assess outcomes and the long-term impact of drug prices. The Markov cohort was utilized for the first time to evaluate the long-term value of pharmaceutical care for young and middle-aged patients with hypertension. The objective of this study was to establish a feasible and cost-effective IPC in the context of the current epidemic situation.

## Materials and methods

### Patients’ characteristics

The Institutional Review Board has approved this study. All patients have signed an informed consent form. From March 1,2021 to February 28, 2022, 97 patients with hypertension were enrolled at Fuwai Yunnan Cardiovascular Hospital in Kunming, China. The enrolled patients had not previously participated in any other national or community health support program related to their hypertensive health issues. Initially, all patients who met the criteria were willing to participate in the program. The duration of the treatment phase was one year. The inclusion criteria, according to the enrollment criteria, were as follows: 1) patients who were young and middle-aged (18 ~ 65 years old); 2) essential hypertension based on the Chinese Hypertension Guidelines for 2020 [21]; 3) patients who had been taking antihypertensive medication for at least two months; 4) patients who were proficient in using communication tools, able to participate in online education, and capable of completing an online questionnaire. The exclusion criteria included: 1) patients who had co-morbidities other than hypertension; 2) patients with severe heart, lung, liver, or renal diseases; 3) patients with communication problems or other impairments such as visual or hearing impairment.

### Internet-based pharmaceutical care (IPC)

**Fig. 1:**
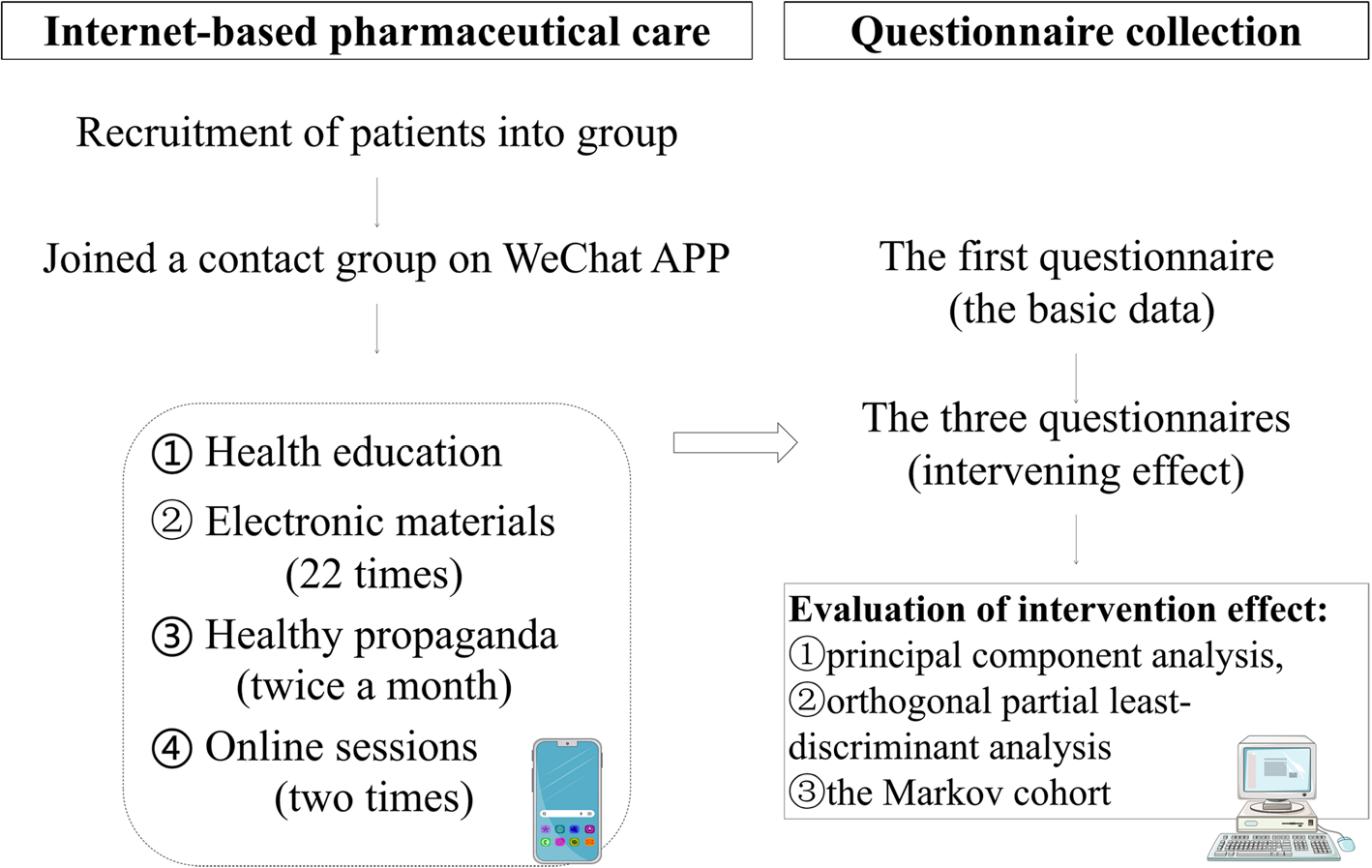
A flow chart of Internet-based pharmaceutical care model in one year

The IPC, based on the PICO model (Problem, Intervention, Comparison, Outcomes), was primarily conducted by pharmacists. This patient-centred pharmaceutical care was divided into four key steps. Firstly, problems were identified through questionnaires. Secondly, the pharmacist made timely interventions. Thirdly, blood pressure and questionnaire results were compared before and after the intervention. Finally, the effect of the pharmacist’s intervention was evaluated. This patient-centered pharmaceutical care consisted of two parts: health education and questionnaire evaluation, as illustrated in Fig. 1. The questionnaire was designed and constructed based on relevant literature and the results of interviews with patients (unpublished data) [22]. Prior to this study, it underwent an evaluation of reliability and validity in 80 patients in our hospital (unpublished data). The questionnaire was divided into three parts (Table 1): patient information (including blood pressure), medication compliance (Q1-Q14), and living habits (Q15-Q25). Patients score themselves from 1 to 5 based on their actual situation during the last month with 1 indicating no or very little time; 2 indicating a small part of the time; 3 indicating half the time; 4 indicating most of the time; 5 indicating all the time. Reverse scoring questions(Q1, Q6-Q13) were processed before participating in the statistical analysis calculation of scores. Some studies have pointed out that the increase in time may lead to the reliability of questionnaire results. According to the estimation of the time for patients to fill in the questionnaire in the early stage, 10 min is acceptable [23, 24]. Software was used to capture the time taken for patients to complete the questionnaire. The completion time for the simulation test had to be less than 10 min.

**Table 1:**
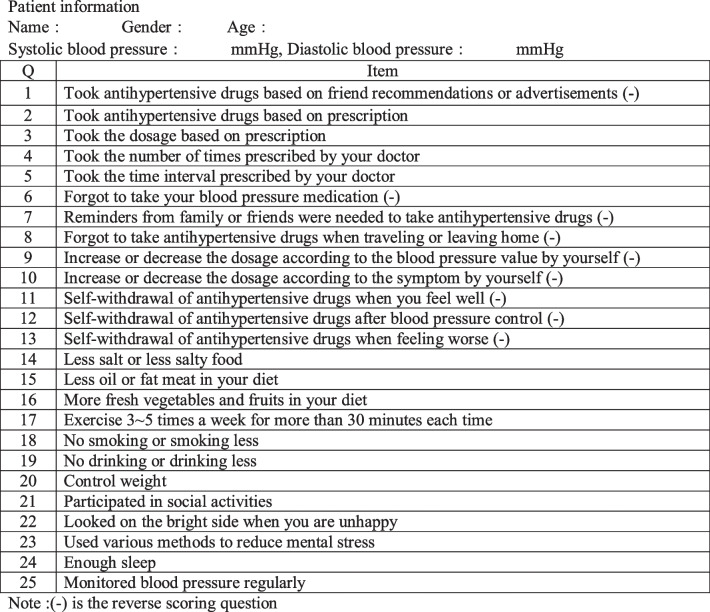
Internet-based scale of hypertension compliance questionnaire

The patients completed the first questionnaire upon enrollment as the basic data. At the same time, they were added to a pharmacist contact group on the WeChat app, where they could receive advice if they encountered any issues with their medications. The basic knowledge of hypertension management methods was announced twice a month to the contact group. Additionally, two online sessions were conducted with guest physicians over the course of a year to discuss life stress reduction, smoking cessation, and alcohol consumption limitation. Electronic health materials were distributed 22 times, and health management materials were posted once. After the intervention, patients were asked to complete the questionnaire three times (every three months) to evaluate the effectiveness of the IPC. The index for controlling blood pressure was defined as having a systolic blood pressure ≤ 120 and a diastolic blood pressure ≤ 90, measured at home during nighttime [21]. The target blood pressure control rate after one year of intervention was set at least 70% [22].

### Reliability evaluation and analysis of the patient questionnaire

PCA of Simca 14.1 software (Umetrics, Kinnelon, New Jersey) was used to analyze questionnaire data and investigate its reliability. OPLS-DA was used to maximize the separation effect before and after IPC. Variables with importance in projection values > 0.5 or < -0.5 were selected in the S-plot.

### The Markov Cohort

**Fig. 2:**
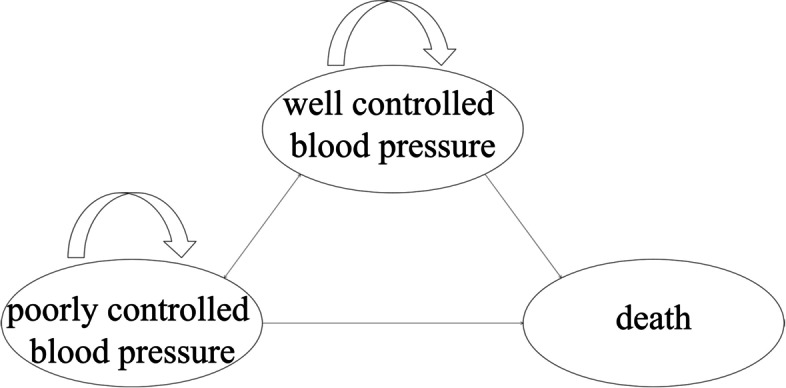
Markov state transition model on Internet-based pharmaceutical care model

Hypertensive disease was defined by three Markov cohort states: poorly controlled blood pressure, well controlled blood pressure, and death (Fig. 2). The Markov cohort period was the same as the pharmacist intervention period (one year). The mean age of the enrolled patients (46.63 years old) was used as the initial value for the simulation. The average life expectancy in 2020 in Yunnan Province, southwest China, was 75.26 years. The average annual cost per patient was calculated by summing up the expenditure used by the intervention patients. The results were compared to the willingness to pay (WTP) value calculated by per capita GDP in Yunnan province in 2021. TreeAge Pro 2011 software (TreeAge Software, Williamstown, Massachusetts) was used to simulate the health transition and calculate the quality adjusted life year (QALY) of hypertensive patients receiving IPC for 28 years.

## Result

### Patients’ characteristics

According to the criteria, 97 young and middle-aged hypertensive patients were enrolled, Of which 96 were followed up (with one patient dropping out during the third questionnaire). The dropout rate was 1% and was due to lack of contact. The characteristics of the 96 patients are presented in Table 2.

**Table 2:** Clinical characteristics of 96 young and middle-aged patients before and after Internet-based pharmaceutical care (IPC)

Characteristic		Male group	Female group
Gender		49	47
Age, year		45.84 ± 9.62	47.33 ± 10.84
Pre-intervention values	Systolic blood pressure, mm Hg	143.04 ± 16.41	133.66 ± 16.63
	Diastolic blood pressure, mm Hg	94.71 ± 12.71	88.77 ± 12.81
	Questionnaire scores	93.78 ± 12.90	99.02 ± 15.17
Post-intervention values	Systolic blood pressure, mm Hg	124.27 ± 14.80	126.91 ± 12.23
	Diastolic blood pressure, mm Hg	85.90 ± 11.68	84.45 ± 10.54
	Questionnaire scores	108.73 ± 9.99	114.77 ± 6.79

### Objective evaluation (blood pressure control)

**Fig. 3:**
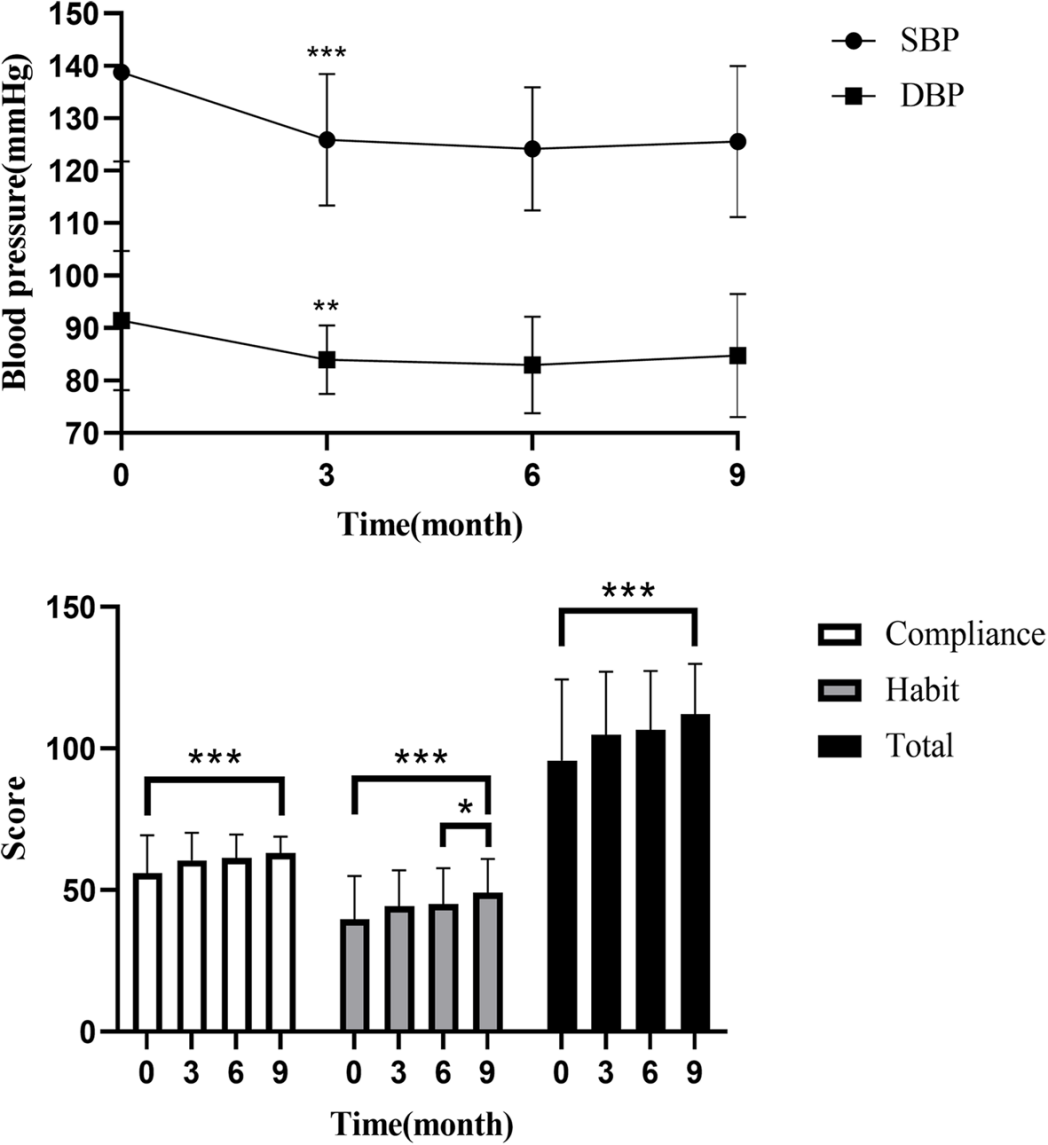
Systolic and diastolic blood pressure (**A**) and questionnaire scores (**B**) before and after Internet-based pharmaceutical care in one year. Statistical analysis was performed using t test. *P < 0.05; **P < 0.01; ***P < 0.001. *SBP* systolic blood pressure, *DBP* diastolic blood pressure

After one year of IPC, the blood pressure control rate increased from 32.29% to 71.88%. The effect of the intervention was pronounced in the first 3 months, with a decrease in systolic blood pressure (SBP) of 13 mmHg on average and diastolic blood pressure (DBP) of 7 mmHg on average. After 6 months of intervention, blood pressure control remained stable (Fig. 3). The systolic blood pressure of female patients decreased by an average of 6.74 mmHg and the systolic blood pressure of male patients decreased significantly by 18.78 mmHg. There was no significant difference in systolic blood pressure and diastolic blood pressure between males (124.27 ± 14.80 mmHg, 85.90 ± 11.68 mmHg) and females (126.91 ± 112.23 mmHg, 84.45 ± 10.54 mmHg) after one year (*P* > 0.05) (Table 2). No drug-related adverse reactions (including dry cough, edema, etc.) were observed during this study.

### Subjective assessment (analysis of questionnaire scores)

The time taken to complete the four questionnaires was: 1) 1.95 ± 2.55 min (after pharmacist training); 2) 6.25 ± 3.19 min; 3) 5.03 ± 2.66 min; 4) 4.20 ± 3.07 min.

#### The credibility of the questionnaire

Simca 14.1 software was used to analyze the reliability of PCA questionnaire data. The results of the first, third, and fourth questionnaires all fell within 99% confidence intervals. The probability that the results of the second questionnaire fell outside the 99% confidence interval (Supplement Fig. 1) was 4.17%, which was within the acceptable range of 10%. The patients whose questionnaire results were outside the confidence interval were NO.1, NO.35, NO.62, and NO.70. The differential contribution plot of No.1 and No.62 patients was Q1-Q4, and the differential contribution plot of No.35 and No.70 patients was Q11-Q13 (Supplement Fig. 2). Pharmaceutical intervention should focus on the related problems of these several patients.

#### The questionnaire score

There were no significant differences in questionnaire scores between the two adjacent groups. There was a difference before and after the intervention (*P* < 0.05) (Fig. 3). The effect of lifestyle intervention was more obvious than that of medication compliance. After one year of intervention, the questionnaire scores increased by an average of 14.96 points for male patients and 15.74 points for female patients, which was statistically significant (*P* < 0.0001).

#### OPLS-DA analysis and s-plot

**Fig. 4:**
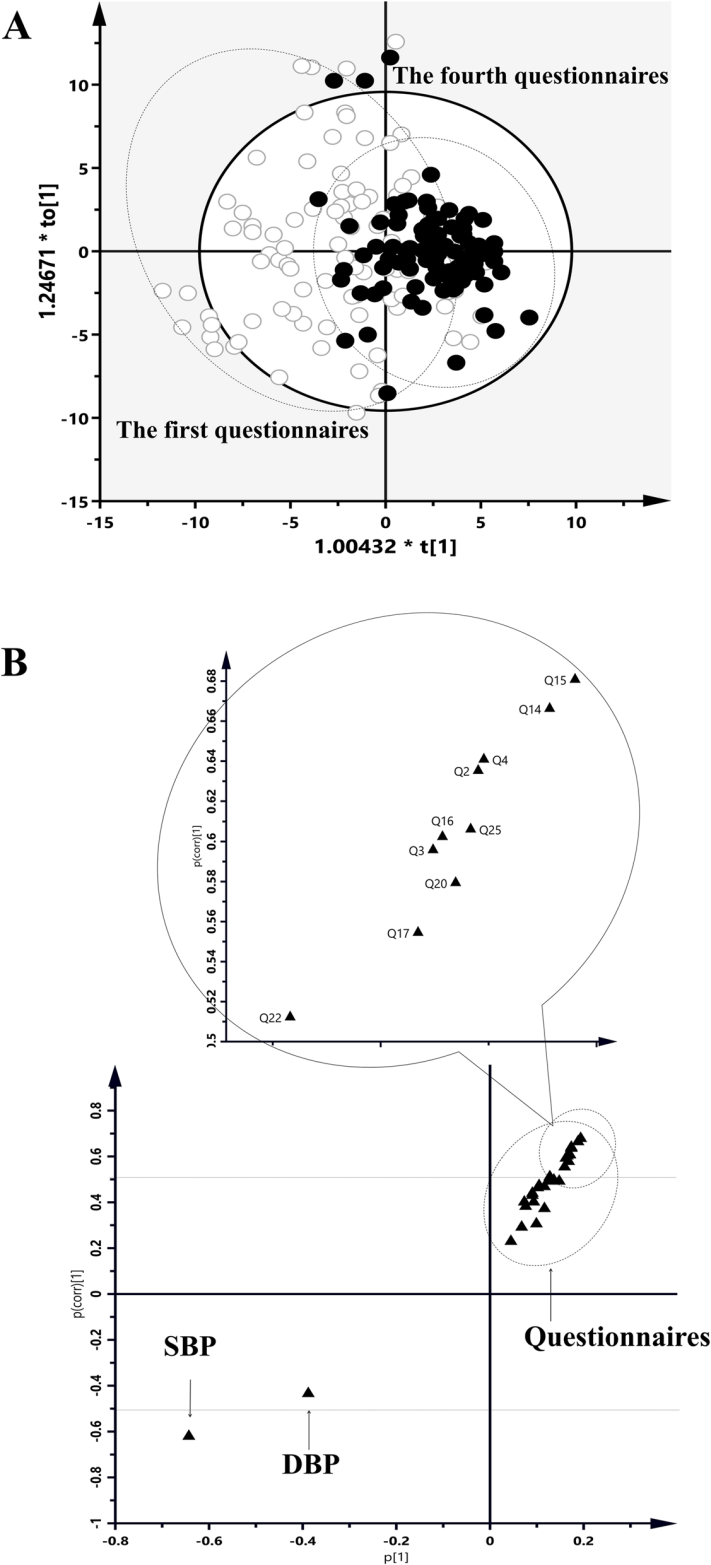
Analysis in the first questionnaires and the first questionnaires by OPLS-DA score plots (**A**) and S-plot with comparison of 10 difference questions (**B**) (●, the first questionnaires; ○, the fourth questionnaires; 10 differential questions are labeled in S-plots). Statistical analysis was performed using t test. *P < 0.05; **P < 0.01; ***P < 0.001. *SBP* systolic blood pressure, *DBP* diastolic blood pressure, *Q* Question

The S-plot of the OPLS-DA analysis showed a clear sesparating trend for the distribution of data for the two groups before and after the intervention. The VIP value of systolic blood pressure in the patient was < -0.5, indicating that the patient's systolic blood pressure decreased significantly. A total of 10 questions in the questionnaire had VIP values > 0.5, indicating that the scores improved significantly after the intervention (Fig. 4).

### The Markov cohort

The natural mortality rate in China in 2021 was 7.18‰. The incidence and control rates of hypertension were 27.50% and 11.00%, respectively [25–28]. The mortality rate attributed to hypertension was 64.93%, which was the average annual number of deaths attributed to hypertension (1.8279‰) divided by the average annual number of deaths attributed to cardiovascular disease (2.815‰) [26]. After three months and one year of pharmaceutical intervention, the number of patients with effective blood pressure control was 31 (32.29%) and 69 (71.88%) [28]. The average mortality rate for hypertensive diseases was calculated by the following formula:

$${\mathrm{P}}_{\mathrm{MH}}={\mathrm{P}}_{\mathrm{IR}}\times {\mathrm{P}}_{\mathrm{CDMR}}\times {\mathrm{P}}_{\mathrm{MACD}}\times 100\mathrm{\%}=0.2750\times 0.455\times (182.79/281.5)\times 100\mathrm{\%}=8.12\mathrm{\%}$$

where MH, IR, CDMR, and MACD represent the average mortality rate of hypertension, hypertension incidence rate, cardiovascular disease mortality rate, and mortality attributable to hypertension in cardiovascular disease, respectively.

The average age of the enrolled patients was 46.63 years old as the initial value of the Markov cohort. Patient survival without intervention was 13.22 years. After one year of pharmacist intervention, patient survival increased by 18.62 years, and the quality adjusted life year (QALY) increased by 5.40 years. The results of the Markov cohort showed that after 28 cycles (28 years), the probability of uncontrolled hypertension was 11.92%, the probability of controlled hypertension was 31.06%, and the probability of death was 57.02%. The results of the Markov cohort for 96 patients are presented in Table 3.

**Table 3:** Results of the Markov cohort of Internet-based pharmaceutical care model (IPC)

Stage	The number of patients without intervention			The number of patients with IPC		
	Poorly controlled blood pressure	Well controlled blood pressure	Death	Poorly controlled blood pressure	Well controlled blood pressure	Death
0	96	0	0	96	0	0
1	78	10	8	25	63	8
2	67	15	14	24	62	10
3	59	17	20	23	60	13
4	53	19	25	22	59	15
5	48	19	29	22	57	17
6	45	18	33	21	55	19
7	41	18	37	21	54	22
8	39	17	40	20	52	24
9	36	16	44	20	51	26
10	34	15	47	19	49	28
11	32	14	50	18	48	29
12	30	14	52	18	47	31
13	28	13	55	17	45	33
14	27	12	57	17	44	35
15	25	11	59	16	43	37
16	24	11	62	16	42	38
17	22	10	64	16	41	40
18	21	10	65	15	39	41
19	20	9	67	15	38	43
20	19	8	69	14	37	44
21	18	8	71	14	36	46
22	17	8	72	14	35	47
23	16	7	73	13	34	49
24	15	7	75	13	33	50
25	14	6	76	12	32	51
26	13	6	77	12	32	52
27	12	6	78	12	31	54
28	12	5	79	11	30	55

*IPC* Internet-based pharmaceutical care model

### IPC cost evaluation

This study set one-time per capita GDP as the threshold value for willingness-to-pay (WTP). According to official data, the per capita GDP in 2021 was ¥57,700 in Yunnan, southwest China [29]. The total cost of IPC was ¥7,470, which mainly included the cost of professional advice, printing materials, and shipping healthcare materials. The average annual intervention cost per patient was ¥77.80 per patient. The cumulative cost for the 28 cycles of the Markov cohort was ¥470.37. The cumulative cost-effectiveness ratio was ¥87.10 per QALY, which was also below the one-time per capita GDP.

### Sensitivity analyses

The probability of achieving blood pressure control in the study was determined using field data. Since the follow-up time was only one year, the blood pressure control rate was not very accurate. Therefore, a single factor sensitivity analysis was performed for the probability of conversion. With a 10% reduction in the control rate, the deaths rose to 57 after 28 years in the sensitivity analysis of the Markov cohort wherein the control rate was the influencing factor. As the control rate rose, the deaths decreased to 52. Effective interventions could significantly reduce the mortality of patients.

The cost of IPC was ¥7,470. The implications of changes in cost (± 20%) were investigated. The cumulative cost for the 28 cycles of the Markov cohort ranged from ¥378.85 to ¥561.90. This value is well below the per capita GDP of ¥57,700. This result indicates that patients have a higher willingness to pay for IPC.

## Discussion

### PCA analysis, OPLS-DA analysis, and the Markov cohort

All three methods were initially used to evaluate the IPC. For the first time, PCA analysis in combination with OPLS-DA analysis was applied to evaluate questionnaires, demonstrating its ability to quickly filter data, and identify reliable questionnaires. Additionally, the advantages of this method, which enable rapid screening of reliable questionnaires and searching for differences between pre- and post-intervention questionnaires, become more important as the number of patients increases.

The application of the Markov cohort in economic comparisons of treatment regimens is widely recognized [30–33]. This study is the first to evaluate the intervention service itself using the Markov cohort. By varying the probability of blood pressure control before and after intervention, the Markov cohort was used to determine the QALYs of the patients. The QALYs was 5.40 years over 28 cycles for the average age of the enrolled patients (46.63 years old). The youngest patient enrolled in this study was 28 years old. The QALYs gained over 47 cycles at the youngest age was 26.30 years, an increase of 2.27 years compared to the influx at the average age. This result is consistent with the theory that early detection and treatment of hypertension leads to better treatment efficacy [25–37].

### Young and middle-aged patients with hypertension

In this study, young and middle-aged patients used Wechat software to complete the questionnaire, a cost-effective and convenient software that takes advantage of the cell phone and Internet-dependent nature of the young and middle-aged population. Previous studies have suggested that patients' high workloads and busy lifestyles are the main causes of noncompliance (terminal and irregular medication use), which was confirmed in this study. The blood pressure was higher during the fourth visit than at enrollment in five patients. The field report of elephone follow-up revealed that three patients had stopped taking their medication on their own, but after receiving timely education, they are now taking it normally. One patient responded directly to the pharmacist that he was working a lot of overtime during this time of the year and had a bad time off. One patient had unstable blood pressure and did not consult a physician in time [37]. Patients’ occupation was an important confounder that might affect patients’ quality of life as well as their response to antihypertensive medications depending on the stress and exhaustion which can affect blood pressure. With the consideration of the limitations in occupation, the intervention methods in this study might need to be refined. Future research could apply more theories (including the Behavior Change Wheel) and viable educational methods (including Behavior Change Techniques) by introducing more reliable and sophisticated intervention instruments [38].

### Cost-Effectiveness of IPC

With the advent of COVID-19, many countries have conducted similar studies on Internet-based mobile health services [39]. These methods mainly included: voice telephone interventions, SMS reminders, electronic medicine box triggers, mobile APP applications, internet-based detection modes, applications of intelligent sphygmomanometer, and WeChat. The intervention durations ranged from 5 weeks to 2 years [40]. However, none of these studies evaluated the implementation of the Internet-based intervention in a long-term manner. This study was the first attempt to evaluate the long-term impact of pharmaceutical care using the Markov cohort.

Data was collected from a questionnaire of 96 patients conducted by two pharmacists with each pharmacist's management time being limited. The results suggest that there might be an inverse relationship between the number of patients and IPC costs at this stage of the study. To further study, additional patients could be enrolled. However, we were also concerned that the quality of the intervention might decline as the number of patients increases. Therefore, the optimal ratio between pharmacists and patients needs further study.

The one-year blood pressure control rate was 71.88%, which was higher than the expected target (70.00%), suggesting that the IPC method was effective. The compliance of hypertensive patients improved after one-year intervention, and the QALYs increased by 5.40 years. A meta-analysis of 2877 patients who received the intervention for 6.38 ± 3.11 months showed a compliance rate of 75.69 ± 14.07% in patients who received the intervention offline and online (78.23 ± 17.71%) [41]. Interestingly, there was no correlation between patient compliance and the number of interventions or duration of the intervention in the meta-analysis. Further studies are needed to determine whether increasing the duration beyond one year could improve the control rate and increase QALYs.

### Limitations

The names, dose, and dosage regimens of the antihypertensive medications administered to the patients in the intervention were considered important confounders (variables) that could affect the study's outcome [42]. As the patients enrolled had been those who was hospitalized to adjust the type and dosage of their medications, the study used only blood pressure values and questionnaire scores as statistics and did not clearly aggregate patients' medication history, type of medication, or medication dosage. Further, a cross-sectional study could be conducted using a multi-stage stratified sampling method to analyze health characteristics (gender, age group, disease duration, and obesity) [42].

## Conclusion

The IPC method can significantly improve the blood pressure control rate of patients. In the Markov cohort, the effect of IPC could be objectively assessed by changes in blood pressure control rates. Patients had a strong willingness to pay for IPC. Method innovation: 1) questionnaire analysis method relying on PCA and OPLS-DA is an effective method to evaluate the effect of IPC method; 2) the Markov cohort evaluated the effect of pharmaceutical care.

### Supplementary Information

**Additional file 1: Supplement figure 1.** The principal component analysis (PCA) results of the first (A), second (B), third (C), and fourth (D) questionnaires.

**Additional file 2: Supplement figure 2.** Hotelling’s T2 range column plot of the second questionnaires (A) and contribution plot (B) of NO.1, NO.35, NO.62, and NO.70 patients.

## Data Availability

The original contributions presented in the study are included in the article/supplementary material; further inquiries can be directed to the corresponding author.
