# Supplementary figures and images for: Evaluation of Internet-based pharmaceutical care effect on young and middle-aged patients with hypertension by the principal component analysis and the Markov cohort during COVID-19 pandemic
Source: Health Qual Life Outcomes. 2023 Aug 18;21:92. doi: 10.1186/s12955-023-02168-0 (PMC10436640; doi:10.1186/s12955-023-02168-0)

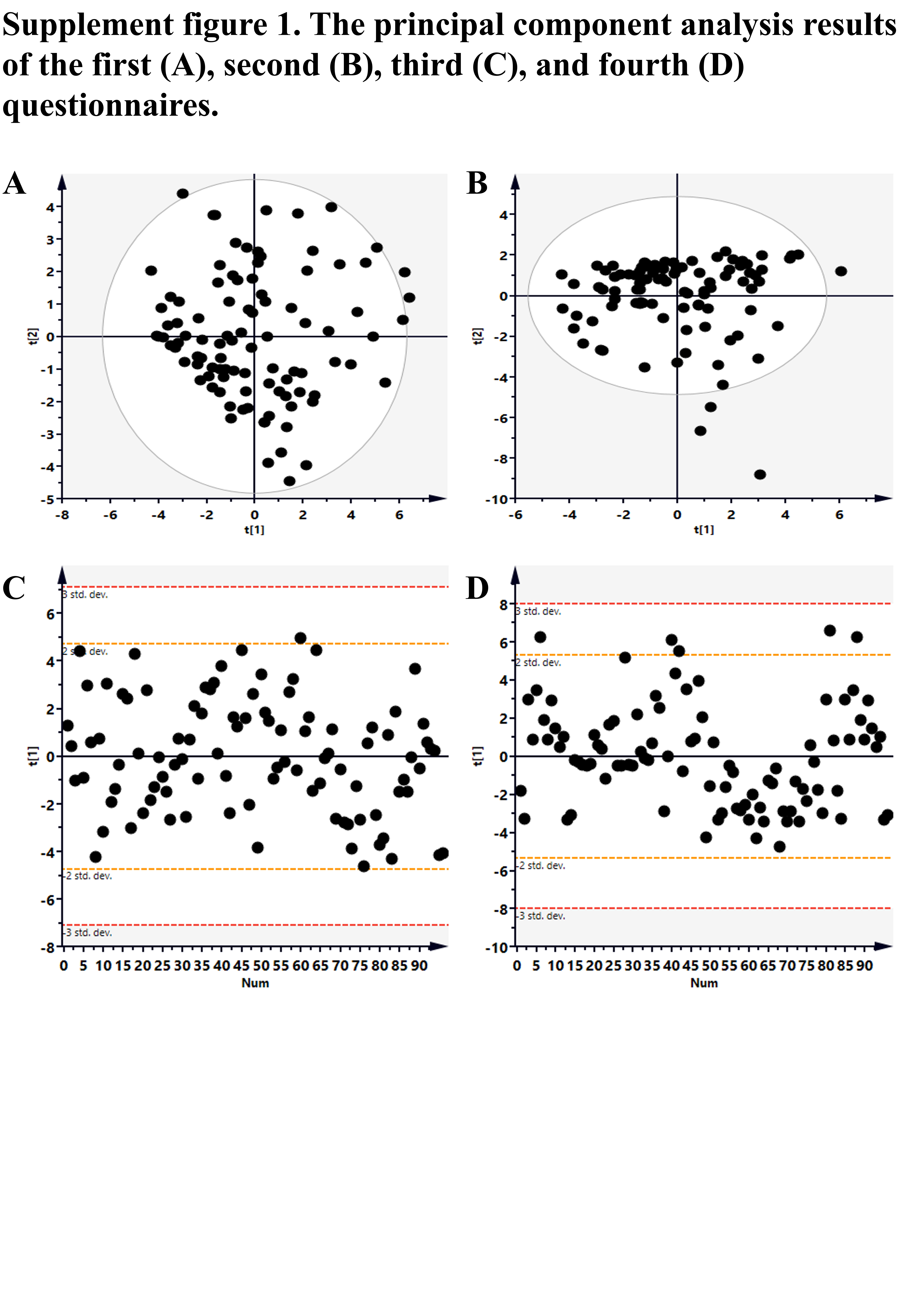

Supplement: Supplementary file 1 — Additional file 1: Supplement figure 1. The principal component analysis (PCA) results of the first (A), second (B), third (C), and fourth (D) questionnaires. [file 12955_2023_2168_MOESM1_ESM.tif]

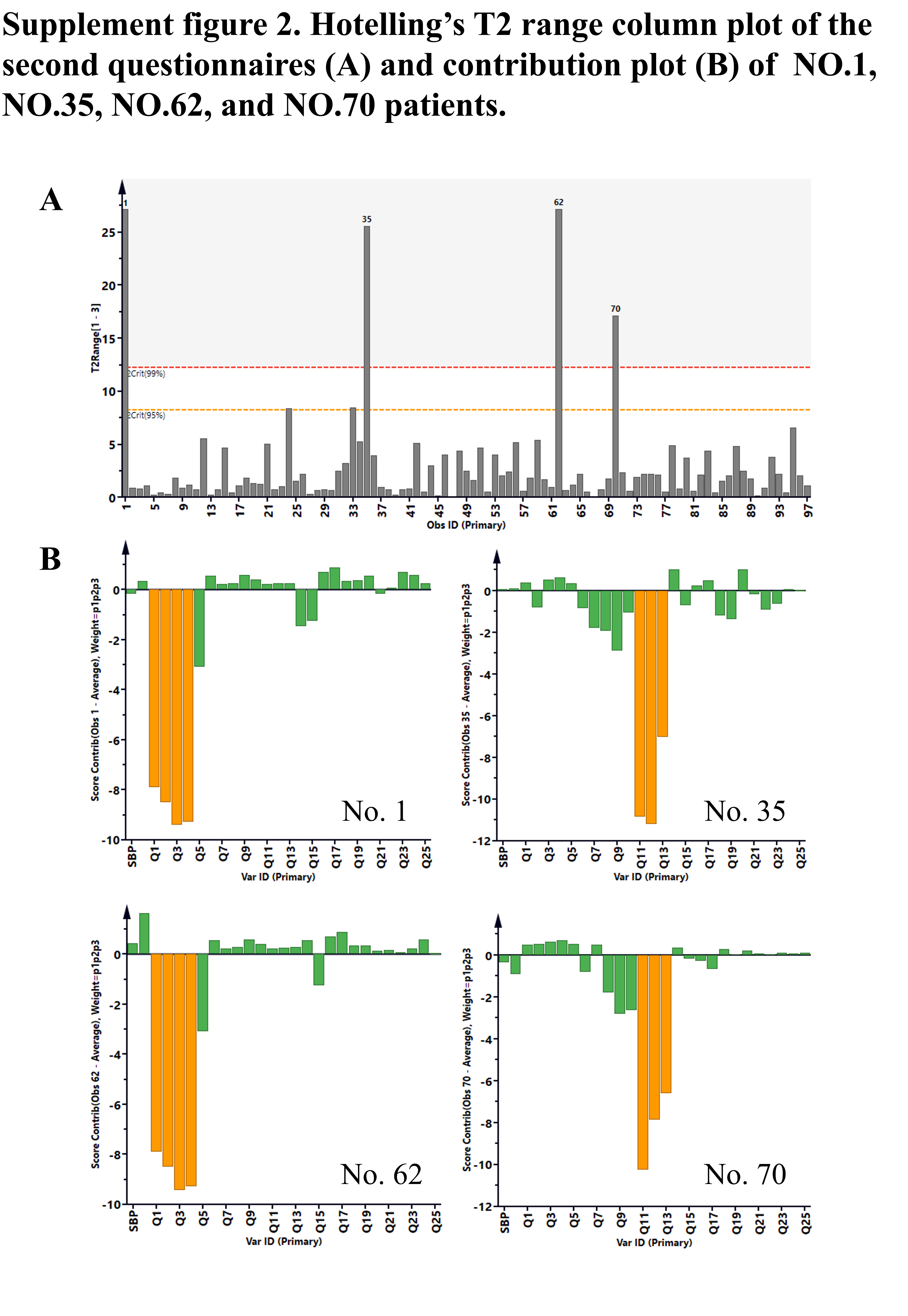

Supplement: Supplementary file 2 — Additional file 2: Supplement figure 2. Hotelling’s T2 range column plot of the second questionnaires (A) and contribution plot (B) of NO.1, NO.35, NO.62, and NO.70 patients. [file 12955_2023_2168_MOESM2_ESM.tif]
